# Supplementary material for: Role and mechanism of NCAPD3 in promoting malignant behaviors in gastric cancer
Source: Front Pharmacol. 2024 Apr 22;15:1341039. doi: 10.3389/fphar.2024.1341039 (PMC11070777; doi:10.3389/fphar.2024.1341039)
Supplement: Supplementary file 11 [file DataSheet2.ZIP › GSEA/Canonical pathways/my_analysis.Gsea.1599462267220/REACTOME_TRANSPORT_OF_INORGANIC_CATIONS_ANIONS_AND_AMINO_ACIDS_OLIGOPEPTIDES.html]

Details for gene set REACTOME\_TRANSPORT\_OF\_INORGANIC\_CATIONS\_ANIONS\_AND\_AMINO\_ACIDS\_OLIGOPEPTIDES[GSEA]

|  || Dataset | filtered\_dataset.sample\_info.cls#WT\_versus\_NCAPD3\_MUT |
| Phenotype | sample\_info.cls#WT\_versus\_NCAPD3\_MUT |
| Upregulated in class | NCAPD3\_MUT |
| GeneSet | REACTOME\_TRANSPORT\_OF\_INORGANIC\_CATIONS\_ANIONS\_AND\_AMINO\_ACIDS\_OLIGOPEPTIDES |
| Enrichment Score (ES) | -0.5014891 |
| Normalized Enrichment Score (NES) | -2.0403051 |
| Nominal p-value | 0.009852217 |
| FDR q-value | 0.020468507 |
| FWER p-Value | 0.077 |
Table: GSEA Results Summary

  

Fig 1: Enrichment plot: REACTOME\_TRANSPORT\_OF\_INORGANIC\_CATIONS\_ANIONS\_AND\_AMINO\_ACIDS\_OLIGOPEPTIDES      
 Profile of the Running ES Score & Positions of GeneSet Members on the Rank Ordered List

  

| SYMBOL | TITLE | RANK IN GENE LIST | RANK METRIC SCORE | RUNNING ES | CORE ENRICHMENT || 1 | 1497 | CTNS | 58 | 0.883 | 0.0494 | No |
| 2 | 23315 | SLC9A8 | 398 | 0.537 | -0.1382 | No |
| 3 | 801 | CALM1 | 424 | 0.518 | -0.1028 | No |
| 4 | 6507 | SLC1A3 | 464 | 0.496 | -0.0797 | No |
| 5 | 6558 | SLC12A2 | 839 | 0.276 | -0.3192 | No |
| 6 | 6520 | SLC3A2 | 878 | -0.290 | -0.3166 | No |
| 7 | 8140 | SLC7A5 | 890 | -0.305 | -0.2930 | No |
| 8 | 54407 | SLC38A2 | 1182 | -0.553 | -0.4446 | Yes |
| 9 | 23657 | SLC7A11 | 1205 | -0.581 | -0.4005 | Yes |
| 10 | 8501 | SLC43A1 | 1296 | -0.690 | -0.3940 | Yes |
| 11 | 57835 | SLC4A5 | 1319 | -0.736 | -0.3340 | Yes |
| 12 | 6509 | SLC1A4 | 1330 | -0.752 | -0.2638 | Yes |
| 13 | 9990 | SLC12A6 | 1379 | -0.922 | -0.2032 | Yes |
| 14 | 6541 | SLC7A1 | 1388 | -0.981 | -0.1080 | Yes |
| 15 | 6519 | SLC3A1 | 1404 | -1.195 | 0.0043 | Yes |
Table: GSEA details [plain text format]

  

Fig 2: REACTOME\_TRANSPORT\_OF\_INORGANIC\_CATIONS\_ANIONS\_AND\_AMINO\_ACIDS\_OLIGOPEPTIDES      
 Blue-Pink O' Gram in the Space of the Analyzed GeneSet

  

Fig 3: REACTOME\_TRANSPORT\_OF\_INORGANIC\_CATIONS\_ANIONS\_AND\_AMINO\_ACIDS\_OLIGOPEPTIDES: Random ES distribution      
 Gene set null distribution of ES for **REACTOME\_TRANSPORT\_OF\_INORGANIC\_CATIONS\_ANIONS\_AND\_AMINO\_ACIDS\_OLIGOPEPTIDES**
